# Artificial Solar Light-Driven APTES/TiO_2_ Photocatalysts for Methylene Blue Removal from Water

**DOI:** 10.3390/molecules27030947

**Published:** 2022-01-30

**Authors:** Agnieszka Sienkiewicz, Paulina Rokicka-Konieczna, Agnieszka Wanag, Ewelina Kusiak-Nejman, Antoni W. Morawski

**Affiliations:** Department of Inorganic Chemical Technology and Environment Engineering, Faculty of Chemical Technology and Engineering, West Pomeranian University of Technology in Szczecin, Pułaskiego 10, 70-322 Szczecin, Poland; agnieszka.sienkiewicz@zut.edu.pl (A.S.); agnieszka.wanag@zut.edu.pl (A.W.); ewelina.kusiak@zut.edu.pl (E.K.-N.); antoni.morawski@zut.edu.pl (A.W.M.)

**Keywords:** photocatalytic water treatment, titanium dioxide, APTES, artificial solar light, methylene blue decomposition

## Abstract

A visible-light photocatalytic performance of 3-aminopropyltriethoxysilane (APTES)-modified TiO_2_ nanomaterials obtained by solvothermal modification under elevated pressure, followed by calcination in an argon atmosphere at 800–1000 °C, is presented for the first time. The presence of silicon and carbon in the APTES/TiO_2_ photocatalysts contributed to the effective delay of the anatase-to-rutile phase transformation and the growth of the crystallites size of both polymorphous forms of TiO_2_ during heating. Thus, the calcined APTES-modified TiO_2_ exhibited higher pore volume and specific surface area compared with the reference materials. The change of TiO_2_ surface charge from positive to negative after the heat treatment increased the adsorption of the methylene blue compound. Consequently, due to the blocking of active sites on the TiO_2_ surface, the adsorption process negatively affected the photocatalytic properties. All calcined photocatalysts obtained after modification via APTES showed a higher dye decomposition degree than the reference samples. For all 3 modifier concentrations tested, the best photoactivity was noted for nanomaterials calcined at 900 °C due to a higher specific surface area than materials calcined at 1000 °C, and a larger number of active sites available on the TiO_2_ surface compared with samples annealed at 800 °C. It was found that the optimum concentration for TiO_2_ modification, at which the highest dye decomposition degree was noted, was 500 mM.

## 1. Introduction

In the past decades, photocatalysis has been proven to be an effective approach for degrading organic compounds. Due to its advantages, such as good chemical stability and low cost, TiO_2_ as a photocatalyst is widely and successfully used in different fields, such as water and wastewater treatment, air cleaning, automotive, buildings materials, agriculture, and antiseptics production [1,2]. Nevertheless, it requires the use of relatively high photon energy for its activation (3.23 eV for the most photoactive anatase phase). For this reason, many methods have been proposed to reduce the band gap energy of TiO_2_ [3,4,5]. Among them, doping TiO_2_ with non-metals, such as nitrogen or carbon, is frequently described as one of the most effective ways to enhance its photoactivity under visible light [6,7]. In addition, TiO_2_ doping with non-metals results in changes in the electronic band structure, lowering the band gap energy [8].

Additionally, the beneficial effects of single-, co-, and tri-doping on characteristics, photocatalytic activity, and the possible applications of the doped TiO_2_ have been discussed in various publications over the years [8,9,10,11]. One promising solution for the modification of TiO_2_ with tri-doping is an application of C, N, and Si [12]. Incorporating silicon into the titanium dioxide surface would increase the specific surface area, reduce the particle size, and hinder the anatase-to-rutile phase transition [13,14], while modification with C and N would commonly improve the photocatalysts’ efficiency in sunlight [15,16]. The most recent method involves using organosilane coupling agents to modify the surface of TiO_2_ [17,18]. One of them is 3-aminopropyltriethoxysilane (APTES), which contains one aminopropyl and three ethoxy functional groups attached to the central Si atom [12,19]. APTES/TiO_2_ nanomaterials can be successfully applied in many different fields. Shakeri et al. [20] investigated the self-cleaning ability of ceramic tile surfaces coated with APTES-modified TiO_2_. They noted that the resulting coating was stable, and the surface could effectively photodegrade the pink food dye selected as an organic pollutant. Nadzirah et al. [21] obtained an APTES/TiO_2_ nanoparticle biosensor-based transducer successfully applied as a sensing platform for *E. coli*. Andrzejewska et al. [22] reported the results of TiO_2_ modification with various aminosilanes that was carried out to obtain pigments via adsorption of organic dyes on modified TiO_2_ surface. Lee et al. [23] successfully prepared APTES-modified TiO_2_ materials by simultaneous amination of TiO_2_ nanoparticles in the gas phase synthesis for possible biomedical applications. Bao et al. [24] also proposed a production method of aminosilane-functionalized TiO_2_ nanomaterials. They reported that prepared samples were capable of photocatalytic decolorization of brilliant red X-3B under UV and visible-light irradiation. López-Zamora et al. [25] presented a new method of TiO_2_ modification with organosilane coupling agents to improve the dispersion of the particles in aqueous systems. They observed that APTES/TiO_2_ samples showed better colloidal stability in water than untreated TiO_2_.

The novelty of the present research was in investigating the photocatalytic activity of APTES-modified TiO_2_ nanomaterials under artificial solar radiation. For the first time, photocatalysts were synthesized by solvothermal modification of TiO_2_ in a pressure autoclave at 180 °C followed by calcination in an argon atmosphere in the temperature range from 800 to 1000 °C. A cationic dye methylene blue was chosen as a model organic pollutant of water.

## 2. Results and Discussion

### 2.1. Characterization of the APTES-Modified TiO_2_

#### 2.1.1. XRD Analysis

**Figure 1:**
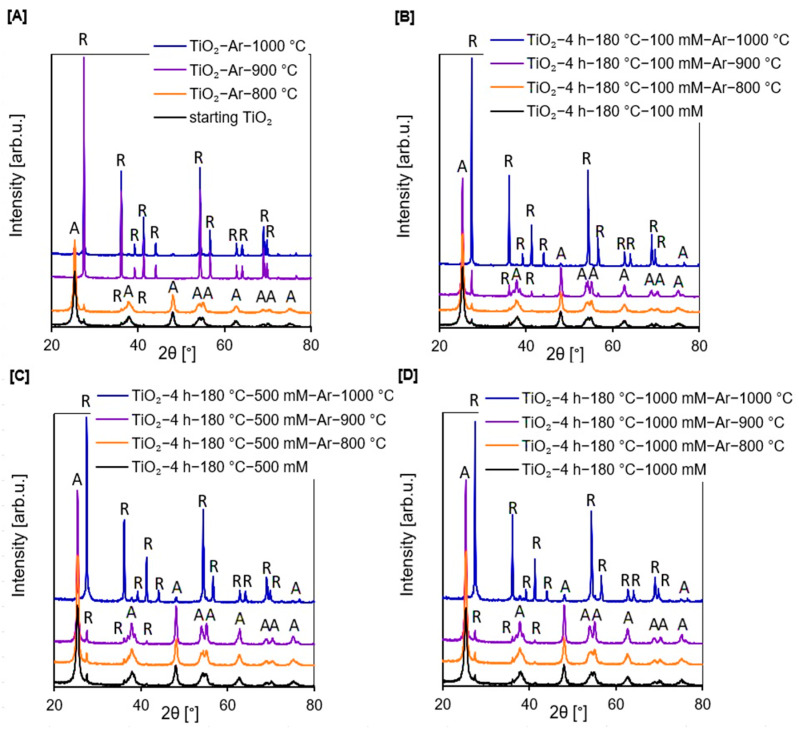
X-ray diffraction patterns of starting TiO_2_, reference photocatalysts (**A**), and APTES/TiO_2_ nanomaterials (**B**–**D**).

According to Figure 1A–D, in the presented X-ray diffraction patterns, all examined photocatalysts except TiO_2_-Ar-900 °C and TiO_2_-Ar-1000 °C showed reflections characteristic for the anatase (04-002-8296 PDF4+ card) and certain reflections characteristic for the rutile phase [26]. Only materials obtained by calcination of the starting TiO_2_ at 900 °C and 1000 °C were characterized exclusively by reflections characteristic for the rutile (04-005-5923 PDF4+ card) [27,28]. The rutile presence in the starting TiO_2_ was due to the addition of rutile nuclei during the raw TiO_2_ pulp production process by the sulphate method [26]. The anatase-to-rutile phase transition starts generally above 600 °C [29]. Therefore, all reference materials consisted of the rutile phase.

Following the data listed in Table 1, the amount of anatase in all non-calcined APTES/TiO_2_ materials was constant at about 96%. Furthermore, it should be noted that, after heating at 900 °C, the APTES-modified TiO_2_ samples still had a very high amount of anatase phase (87–94%). Moreover, even nanomaterials calcined at 1000 °C did not consist exclusively of the rutile phase, as they contained 6–16% anatase phase. The silicon and carbon derived from APTES contributed to the successful delay of the anatase-to-rutile phase transformation during heating [14,30,31,32], so that the higher the concentration of the used modifier, the better effect of phase transformation inhibition. The crystallite size of both polymorphous forms of TiO_2_ grew with increasing calcination temperature (see Table 1); although, when comparing the crystallites size of nanomaterials heated at the same temperature with and without the modifier, the crystallites of both rutile and anatase were smaller for the APTES/TiO_2_ materials with respect to calcined reference samples. For instance, the crystallite size of anatase for TiO_2_-Ar-800 °C was >100 nm, while for TiO_2_-4 h-180 °C-1000 mM-Ar-800 °C equalled merely 19 nm.

The results reported by Xu et al. [13], Okada et al. [30], and Cheng et al. [33] were consistent with ours, and showed that, with the addition of Si to TiO_2_ causes, during thermal modification, the increase in crystallite size of both polymorphous forms of TiO_2_ was effectively inhibited. According to Wu et al. [34], replacement of surface hydroxyl groups prior to calcination stage with another functional group that does not condense like –OH, such as methyl siloxyl surface group, and can produce small secondary phase particles, results in inhibition of grain boundary broadening at elevated temperatures.

**Figure 2:**
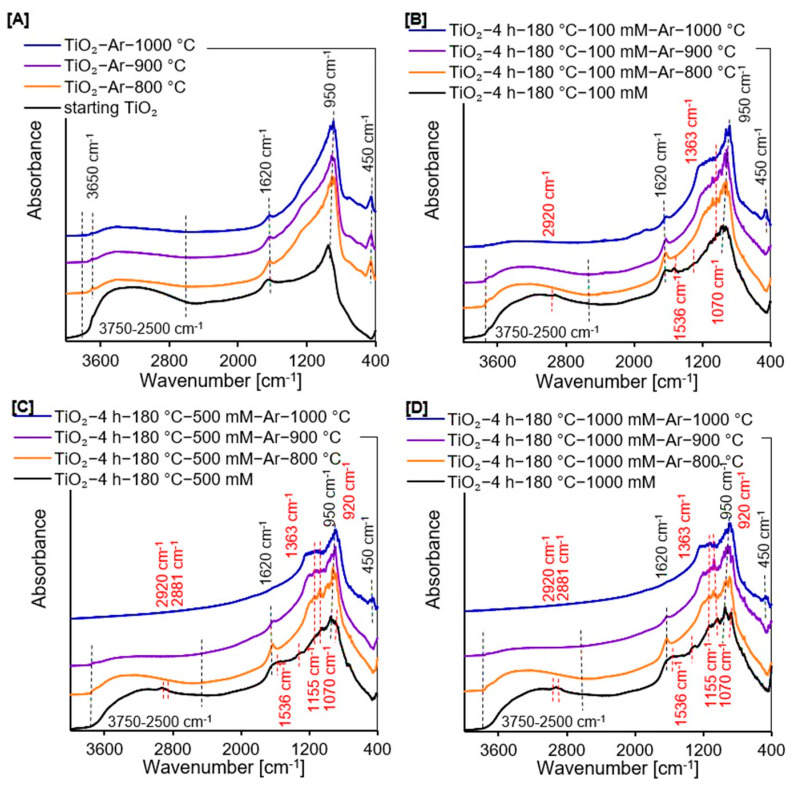
Diffuse reflectance Fourier transform infrared spectra of starting TiO2, reference photocatalysts (**A**), and APTES/TiO2 nanomaterials (**B**–**D**).

The FT-IR/DRS measurements (see Figure 2A–D) confirmed the presence of silicon groups on the surface of APTES/TiO_2_ nanomaterials after annealing, which could suppress the increase in crystallite size compared with the reference samples without silicon groups.

#### 2.1.2. DRIFTS Measurements

The FT-IR/DRS measurements were used to identify the surface characteristics of all prepared samples. All spectra shown in Figure 2A–D exhibited certain peaks characteristic of TiO_2_-based photocatalysts. A strong band located at 950 cm^−1^ is ascribed to the self-absorption of titania [35]. The narrow band at around 1620 cm^−1^ and a wide band from 3750 cm^−1^ to 2500 cm^−1^ attributed to the molecular water bending modes and stretching vibrations of surface –OH groups [14,35,36], respectively, which were observed for starting TiO_2_, all non-calcined APTES-modified TiO_2_, as well as for the reference materials and APTES/TiO_2_ photocatalysts heated below 1000 °C. All reference samples also exhibited the low intensity band at 3650 cm^−1^, ascribed to the stretching mode of various free –OH groups. This implies that the elimination of adsorbed water molecules followed by the removal of bridged –OH groups result in the formation of free –OH groups [37,38]. Moreover, enhancement of the annealing temperature caused a reduction in the intensity of these three aforementioned bands because of alterations in the amount of surface hydroxyl groups [39,40]. Additionally, for all reference materials and APTES-modified TiO_2_ calcined at 1000 °C, a band located around 450 cm^−1^ attributed to the rutile phase was observed [41,42]. Several new characteristic bands from APTES were noted in the spectra presented in Figure 2C,D, indicating that the synthesis of new nanomaterials utilizing the solvothermal method was carried out successfully. The low-intensity bands at around 2881 cm^−1^ and 2920 cm^−1^ belong to the asymmetric and symmetric stretching vibration of alkyl groups [19,20,36,43]. The asymmetric –NH_3_^+^ deformation modes were noticed at 1552 cm^−1^ [19,39,44]. The next low-intensity band located at 1363 cm^−1^ falls in the characteristic region of C−N bonds [19,45]. In addition, the bands at about 1155 cm^−1^ and 1070 cm^−1^ correspond to the Si−O−Si stretching vibrations and Si–O–C stretching mode, respectively [19,46,47]. Furthermore, the bands located between 960 cm^−1^ and 910 cm^−1^ are characteristic for the stretching vibrations of Ti–O–Si bonds. However, the band recorded at around 920 cm^−1^ suggests that the condensation reaction occurred between silanol and surface –OH groups [36,48]. For all APTES/TiO_2_ photocatalysts, bands characteristic for APTES assigned to alkyl groups, –NH_3_^+^ and C–N bonds did not occur after calcination. These groups were not permanently bonded to the TiO_2_ surface. Therefore, annealing at high temperature contributed to the destruction of these bonds.

#### 2.1.3. BET Measurements

**Figure 3:**
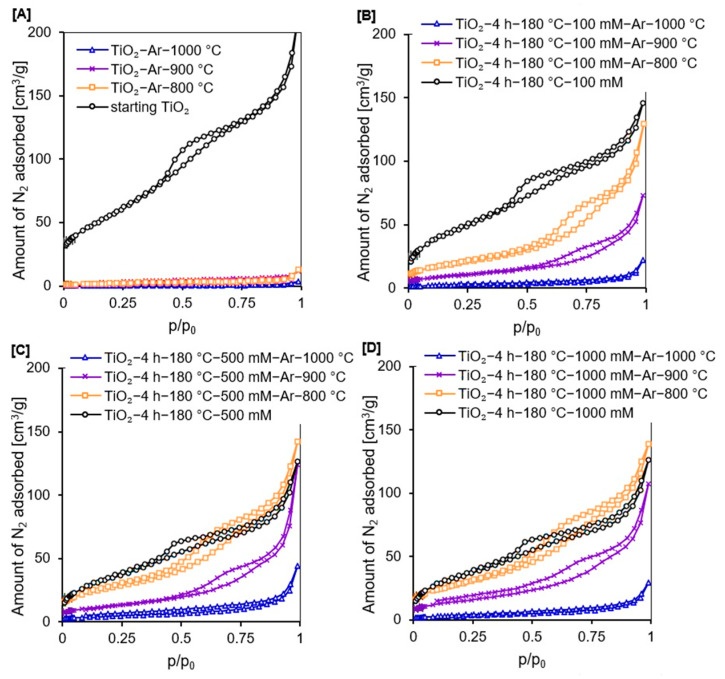
Adsorption–desorption isotherms of starting TiO_2_, reference photocatalysts (**A**), and APTES/TiO_2_ nanomaterials (**B**–**D**).

In Figure 3A–D, the adsorption–desorption isotherms of all the prepared materials are presented. Based on the IUPAC classification, all reference samples and APTES/TiO_2_ nanomaterials calcined at 1000 °C showed a type II isotherm typical for non-porous samples [49]. The other prepared photocatalysts exhibited a type IV isotherm specific for mesoporous materials, and they also showed the H3 type of hysteresis loops [49,50]. The isotherms revealing type H3 do not show limiting adsorption at high *p*/*p*_0_ value and have specific desorption shoulders and lower closure points [49,50,51].

Confirmation of the observations that all calcined reference materials and APTES-modified TiO_2_ heated at 1000 °C were non-porous materials, while all other photocatalysts were mesoporous materials with a small proportion of micropores, as shown by the data presented in Table 1. After modification with APTES, a significant decrease in the total pore volume and specific surface area was observed. Moreover, the higher the concentration of the organosilane modifier, the greater the reduction in the S_BET_ and V_total_. For example, in comparison to the starting TiO_2_, the S_BET_ of TiO_2_-4 h-180 °C-1000 mM decreased by 86 m^2^/g and V_total_ by 0.196 cm^3^/g. Cheng et al. [52] noted that the specific surface area of APTES-modified TiO_2_ was smaller than that of the unmodified photocatalyst due to the coating of modifier on the surface of P25 TiO_2_ nanoparticles. Zhuang et al. [53] reported that the S_BET_ and the pore volume were smaller for APTES/TiO_2_ materials than for untreated TiO_2_ because APTES molecules could penetrate the pores of TiO_2_, leading to a reduction in both S_BET_ and V_total_. Additionally, Hou et al. [54] observed that as the concentration of APTES increases (over 2 wt.%), both the specific surface area and pore volume drastically decrease, due to the formation of a thick coating layer on the TiO_2_ surface, thus, blocking the access of adsorption gas to pores.

After calcination, a significant decrease in S_BET_ and V_total_ was reported for all photocatalysts (see Table 1) due to the increase in crystallites size of the rutile and anatase phase and sintering of nanomaterials particles [55]. However, for the samples modified with APTES, the observed decrease was significantly lower than for the calcined reference materials prepared at the same temperature due to the effective inhibition of the anatase-to-rutile phase transition and the growth of the crystallites size of both TiO_2_ polymorphous forms [13]. For example, for the TiO_2_-Ar-900 °C, the S_BET_ was 3 m^2^/g and V_total_ equalled 0.008 cm^3^/g, while for the TiO_2_-4 h-180 °C-1000 mM-Ar-900 °C the S_BET_ and V_total_ were 55 m^2^/g and 0.166 cm^3^/g, respectively. Moreover, the higher concentration of APTES used for modification, the better the inhibition of the crystallite size growth and, thus, the larger the specific surface area of the obtained nanomaterials [14]. So, for TiO_2_-4 h-180 °C-100 mM-Ar-800 °C the S_BET_ was 70 m^2^/g, for TiO_2_-4 h-180 °C-500 mM-Ar-800 °C it was 95 m^2^/g, while for TiO_2_-4 h-180 °C-1000 mM-Ar-800 °C it equalled 104 m^2^/g.

#### 2.1.4. UV-Vis Diffuse Absorbance Spectroscopy

**Figure 4:**
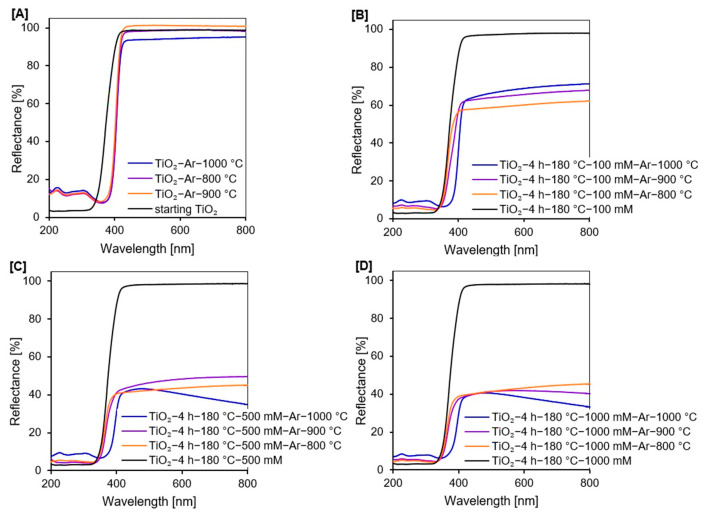
UV-Vis diffuse reflectance spectra of starting TiO_2_, reference photocatalysts (**A**), and APTES/TiO_2_ nanomaterials (**B**–**D**).

From the UV-Vis/DR spectra of all tested photocatalysts, presented in Figure 4A–D, it was noted that starting TiO_2_, reference materials, and all non-calcined APTES/TiO_2_ samples showed the typical absorption in the UV region because of the intrinsic band gap absorption of titanium [56]. However, after calcination, the reflectance of all examined nanomaterials decreased with the increase in the heating temperature due to the color change of photocatalysts from white (non-calcined samples), through to grey (materials calcined at 800 °C), to dark grey (semiconductors modified above 800 °C) [57,58]. The change of color was related to the presence of carbon in the studied samples. Additionally, the spectra of all reference samples and APTES-modified TiO_2_ calcined at 1000 °C showed the absorption peak from 200 to 400 nm with the maximum at 226 and 305 nm. The absorption band at around 305 nm is associated with the charge transfer from O^2−^ to Ti^4+^, related to the excitation from the valence to the conduction band [59,60,61]. After calcination, there was a red shift of the absorption edge towards visible light. The increased absorption was most likely due to the presence of rutile phase, which has an intrinsically smaller band gap energy compared with the pure anatase phase [62,63]. Moreover, the intensity of these bands decreased with increasing concentration of modifier due to the delay of the anatase-to-rutile phase transformation.

According to the band gap energy values of all the studied samples shown in Table 2, it was noted that after calcination in an inert atmosphere, the E_g_ of starting TiO_2_ of 3.29 eV decreased to 3.03 eV for TiO_2_-Ar-800 °C and TiO_2_-Ar-900 °C samples and 3.01 eV for TiO_2_-Ar-1000 °C. While for APTES/TiO_2_ nanomaterials significant changes in E_g_ were reported only for photocatalysts calcined at 1000 °C. This was mainly attributed to the anatase-to-rutile phase transformation [64,65].

#### 2.1.5. SEM and EDX Mapping Analysis

**Figure 5:**
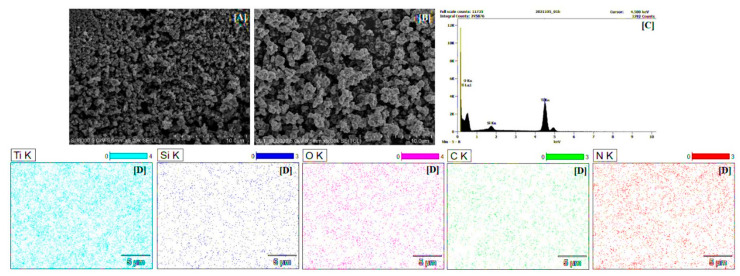
The SEM images of starting TiO_2_ (**A**), TiO_2_-4 h-180 °C-500 mM-Ar-900 °C (**B**), EDX spectrum (**C**), and EDX mappings of TiO_2_-4 h-180 °C-500 mM-Ar-900 °C (**D**).

From the SEM image shown in Figure 5A, it was noted that the starting TiO_2_ morphology was relatively homogenous, but the particles formed aggregates. For TiO_2_-4 h-180 °C-500 mM-Ar-900 °C sample (see Figure 5B), it was observed that functionalization contributed to the increase in aggregates size. The results of EDX mapping analysis, presented in Figure 5C,D, confirmed the presence of Ti and O, as well as Si and C expected after modification, and exhibited that all studied elements were uniformly dispersed on the TiO_2_ surface. The results of EDX mapping analysis are the average of measurements taken at 5 different points. As expected, it was noted that the silicon content increased with an increasing amount of APTES. Thus, the TiO_2_-4 h-180 °C-100 mM-Ar-900 °C sample contained 1.25 wt.% Si, while TiO_2_-4 h-180 °C-500 mM-Ar-900 °C and TiO_2_-4 h-180 °C-1000 mM-Ar-900 °C had 2.13 and 2.26 wt.% Si, respectively.

#### 2.1.6. Carbon and Nitrogen Content Analysis

Based on the carbon and nitrogen content analysis (see Table 2), the existence of C and N in the non-calcined APTES/TiO_2_ materials confirmed the APTES presence in the samples received after functionalization of starting TiO_2_. As expected, it was also observed that the higher the amount of modifier, the higher the content of the analyzed elements [12,66]. Moreover, it was noted that the quantity of carbon and nitrogen reduced drastically after calcination and kept decreasing with increasing temperature of modification because of the N- and C-containing functional groups decomposition and removal them from the photocatalysts surface [35,67]. Unfortunately, the N content in the APTES/TiO_2_ nanomaterials annealed above 800 °C was below the detection level of the device. The results derived from the C and N content analysis agreed with the data obtained from FT-IR/DR spectra (see Figure 2B–D), which demonstrated a significant decrease in the amount of both analyzed elements in APTES-modified TiO_2_ after the calcination. The presence of 0.18 wt.% of nitrogen could be explained by the preparation procedure of the starting TiO_2_ involving pretreatment with ammonia water, used to remove residual sulfuric acid from the crude TiO_2_ slurry produced by the sulfate method [68].

#### 2.1.7. Zeta Potential Measurements

The zeta potential measurements confirmed the change of surface character from positively to negatively charged after APTES modification (see Table 2). For the reference materials, the change in the TiO_2_ surface charge was most likely related to the anatase-to-rutile phase transformation and total transition to rutile phase. Our observations were consistent with Haider et al. [69], Pinheiro Pinton et al. [70], and Chellappah et al. [71], who reported that the pure rutile exhibits negative zeta potential values. In the case of organosilane/TiO_2_ nanomaterials, Talavera-Pech et al. [72] and Goscianska et al. [73] observed that the cationic amino groups from aminosilane readily link the TiO_2_ surface groups resulting in the positively charged surface. However, the FT-IR/DR spectra shown in Figure 2B–D, and the reduction in nitrogen content (see Table 2), indicated that amino groups were not present on the TiO_2_ surface after calcination. The silicon groups were mainly found on the calcined APTES/TiO_2_ surface. Li et al. [74], Ferreira-Neto et al. [75], and Worathanakul et al. [76] noted that silica-modified titanium dioxide materials were characterized by a negative value of zeta potential.

### 2.2. Adsorption and Photocatalytic Studies

**Figure 6:**
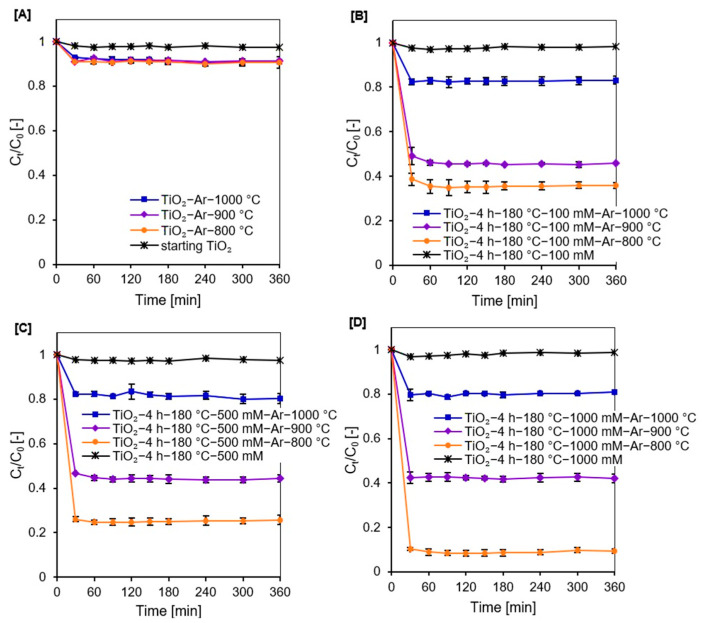
Methylene blue adsorption degree on the surface of starting TiO_2_, reference nanomaterials (**A**), and APTES/TiO_2_ photocatalysts before and after calcination (**B**–**D**).

Before studying the photocatalytic activity of the prepared samples, tests were conducted to establish the adsorption–desorption equilibrium at the photocatalyst–dye interface. The results are presented in Figure 6A–D. For all examined nanomaterials, equilibrium was reached after 60 min. It was also observed that calcination enhanced the adsorption abilities of the obtained materials. From the zeta potential values (see Table 2), it can be concluded that transformation of TiO_2_ surface charge from positive to negative after annealing increased the adsorption abilities of the tested samples. The negatively charged semiconductor surface has a higher potential of contact with the positively charged methylene blue molecules, due to the attractive electrostatic interactions [77,78,79]. Calcined APTES/TiO_2_ nanomaterials showed clearly dissimilar adsorption degrees of the methylene blue compound; although, they demonstrated similar zeta potential values (i.e., 19% for TiO_2_-4 h-180 °C-500 mM-Ar-1000 °C but 75% of adsorbed dye for TiO_2_-4 h-180 °C-500 mM-Ar-800 °C). It is generally agreed that adsorption properties are ascribed to larger specific surface area values. In this case, among all calcined APTES/TiO_2_ samples, nanomaterials heated at 800 °C were characterized by the highest S_BET_ area value, i.e., for TiO_2_-4 h-180 °C-1000 mM-Ar-800 °C the value reached 104 m^2^/g, while for TiO_2_-4 h-180 °C-1000 mM-Ar-1000 °C it was only 12 m^2^/g.

**Figure 7:**
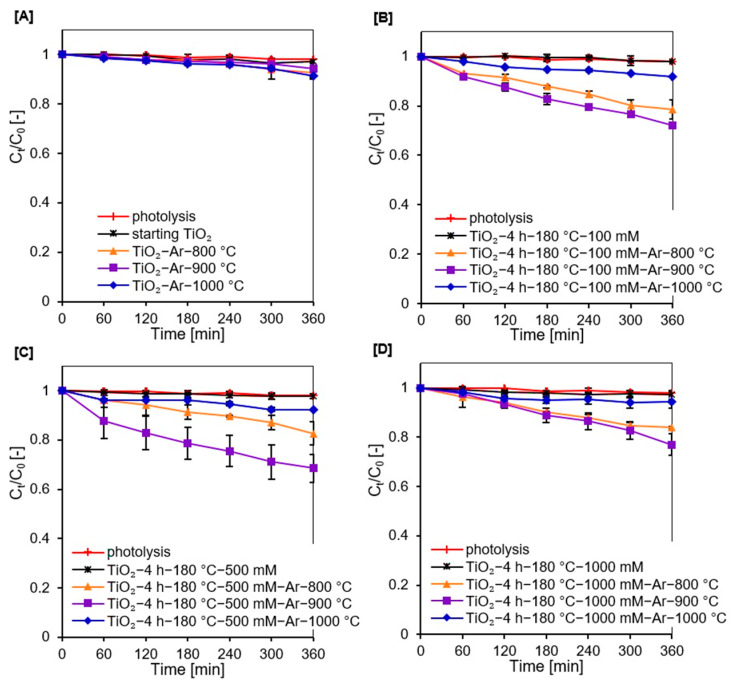
Methylene blue decomposition under artificial solar light irradiation for starting TiO_2_, reference nanomaterials (**A**), and APTES/TiO_2_ photocatalysts before and after calcination (**B**–**D**).

Photodegradation of methylene blue in the presence of APTES/TiO_2_ photocatalysts was investigated under artificial solar light. The results are presented in Figure 7A–D as a plot of C_t_/C_0_ versus irradiation time, where C_0_ is the initial concentration of dye and C_t_ is the concentration at time t.

**Figure 8:**
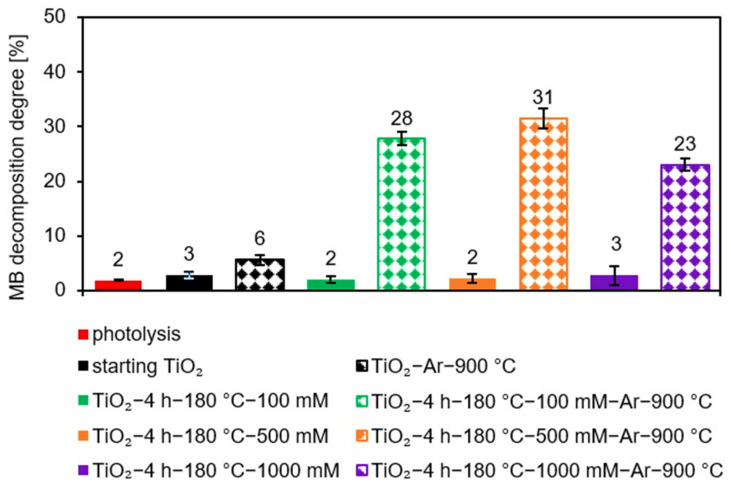
Methylene blue decomposition degree after 360 min of artificial solar light radiation for starting TiO_2_, reference nanomaterial (TiO_2_-Ar-900 °C), and APTES/TiO_2_ samples modified with different amounts of organosilane before and after calcination at 900 °C.

Methylene blue decomposition in the absence of photocatalyst (photolysis test) was negligible (about 2%). Therefore, the effect of photosensitization can be neglected. After thermal modification of starting TiO_2_ no significant changes were observed regarding the improvement of the dye decomposition by the reference materials (see Figure 7A). For the TiO_2_-Ar-900 °C sample, only about 6% methylene blue decomposition degree was achieved (see Figure 8) after 360 min of irradiation.

**Figure 9:**
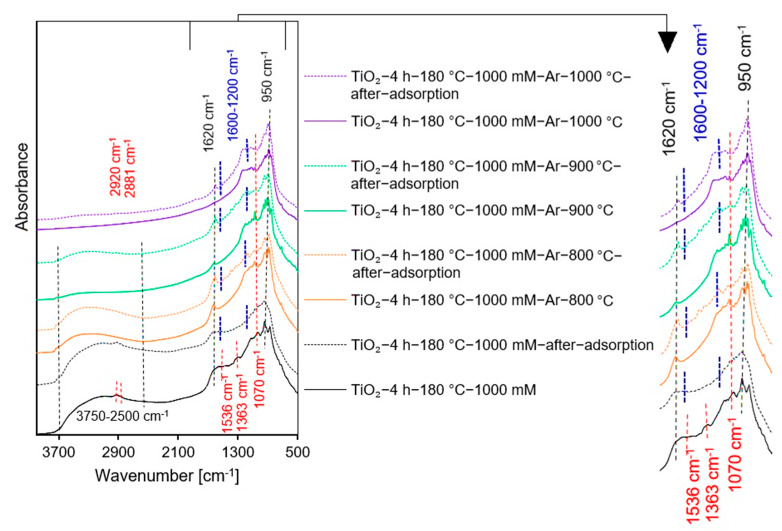
FT-IR/DR spectra of the selected samples before and after adsorption of methylene blue.

For APTES-modified TiO_2_ samples obtained after calcination, a marked improvement in photocatalytic activity was noted. The presence of silicon and carbon in the nanomaterials effectively delays the anatase-to-rutile phase transformation, as well as inhibit the growth of the crystallites size of both TiO_2_ polymorphous forms during calcination [14,30,31]. Thus, compared with the reference samples heated at the same temperature, calcined APTES/TiO_2_ photocatalysts exhibited higher values of specific surface area and pore volume, as well as a larger content of a more active anatase phase, which contributed to a higher methylene blue decomposition degree [12,80]. For all three modifier concentrations used for preparation (100 mM, 500 mM, and 1000 mM), the best methylene blue decomposition degree was received for samples annealed at 900 °C. To explain the highest activity of this nanomaterials, the FT-IR/DR spectra of selected materials were determined after the adsorption process (see Figure 9).

The noticeable enhancement in the intensity of the band localized at 1200–1600 cm^−1^ suggested that carbon deposits from methylene blue appeared on the photocatalyst surface after adsorption, which strongly limited the photocatalytic efficiency [81,82]. Although APTES-modified TiO_2_ photocatalysts calcined at 800 °C showed the highest specific surface area and pore volume, they also showed the highest dye adsorption. As a result, the highest number of active sites on the TiO_2_ surface was blocked by methylene blue molecules. Since presumably at high dye concentrations, the generation of hydroxyl radicals on the photocatalyst surface is limited due to the active sites being covered by dye ions, leading to a decrease in activity [83]. Photocatalysts heated at 1000 °C were characterized by very small specific surface area and pore volume. They also contained a higher amount of rutile than the anatase phase resulting the low photoactivity [84]. Therefore, TiO_2_ modified with APTES calcined at 900 °C showed the highest methylene blue decomposition degree. Furthermore, at a constant annealing temperature of 900 °C, after 360 min of artificial solar light radiation, the methylene blue degradation degree was 23%, 28%, and 31% for nanomaterials modified with 1000 mM, 100 mM, and 500 mM, respectively. Although the TiO_2_-4 h-180 °C-1000 mM-Ar-900 °C photocatalyst exhibited a larger specific surface area than the TiO_2_-4 h-180 °C-500 mM-Ar-900 °C and TiO_2_-4 h-180 °C-100 mM-Ar-900 °C samples, it showed the highest dye adsorption degree. For the TiO_2_ modified with 1000 mM of APTES, the methylene blue adsorption degree was 59%, while for 500 mM was 55%, and for 100 mM it equaled 53%. Therefore, most of active sites were blocked by adsorbed dye molecules on the surface of the TiO_2_-4 h-180 °C-1000 mM-Ar-900 °C sample, resulting in a decrease in photocatalytic activity. Besides the confirmed influence of physicochemical properties on photocatalytic efficiency of the prepared semiconductors, the crucial role of adsorption process was also proved. Considering the highest photoactivity and economic aspects according to which the less modifier the better, TiO_2_-4 h-180 °C-500 mM-Ar-900 °C was selected as the most prospective material.

## 3. Experimental

### 3.1. Materials and Reagents

All photocatalysts were obtained based on the crude TiO_2_ slurry, delivered from the chemical plant Grupa Azoty Zakłady Chemiczne “Police” S.A. (Police, Poland). Before modification, raw TiO_2_ pulp was pre-treated to reach a pH of 6.8. This step was described in detail in our previous article [26]. The received sample was denoted as starting TiO_2_. The modifier of the starting TiO_2_ was 3-aminopropyltriethoxysilane (APTES, ≥98%) from Merck KGaA (Darmstadt, Germany). Ethanol from P.P.H. “STANLAB” Sp.J., (96%, Poland) was utilized as a solvent of APTES. For photocatalytic activity tests, the methylene blue (Firma Chempur^®^, Piekary Śląskie, Poland) was used as a model organic water pollutant.

### 3.2. Preparation Procedure of APTES/TiO_2_ Nanomaterials

The APTES-modified TiO_2_ nanomaterials were prepared via the solvothermal method and calcination process. To modify the TiO_2_ surface, various amounts of modifier were used and the concentrations of APTES in the solvent were 100, 500, and 1000 mM. In the first step, 5 g of starting TiO_2_ was mixed with 25 mL solution of APTES and modified in a pressure autoclave at 180 °C for 4 h, providing continuous stirring at 500 rpm. Next, the obtained suspension was rinsed with ethanol and distilled water to remove all remaining chemicals. Then, the material was dried in a lab dryer for 24 h at 105 °C. The obtained samples were denoted as TiO_2_-4 h-180 °C-XmM, where X is the concentration of modifier in a solvent. In the second stage, the photocatalyst was annealed in an argon atmosphere (purity 5.0, Messer Polska Sp. z o.o., Poland). The calcination process was carried out in a range of temperatures from 800 to 1000 °C, where Δt = 100 °C. The quartz crucible with the resulting material was inserted into a quartz tube in the central section of the GHC 12/900 horizontal furnace (Carbolite Gero, Ltd., UK). Before heating, Ar was run through a tube for 30 min to eliminate residual air. Afterwards, the furnace was heated to the desired temperature at an Ar flow rate of 180 mL/min, with a calcination time of 4 h. Next, the furnace cooled gradually to room temperature. Samples received after annealing of starting TiO_2_ in the inert gas atmosphere were named reference materials, denoted as TiO_2_-4 h-Ar-Y °C, while APTES-modified TiO_2_ received after calcination were denoted as TiO_2_-4 h-180 °C-XmM-Ar-Y °C, where Y is the temperature of annealing.

### 3.3. Characterization of Photocatalysts

The X-ray powder diffraction analysis (Malvern PANalytical B.V., Almelo, the Netherlands, utilizing Cu Kα radiation (λ = 1.54056 Å), applied to determine the crystalline structure of the prepared samples. The PDF-4+ 2014 International Centre for Diffraction Data database was used to specify the phase composition (for rutile: 04-005-5923 PDF4+ card; for anatase: 04-002-8296 PDF4+ card) and to calculate the mean crystallites size the Scherrer’s equation was used. The spectrometer FT-IR-4200 (JASCO International Co. Ltd., Tokyo, Japan), fitted with DiffuseIR accessory (PIKE Technologies, USA), was used to detect the functional groups on the surface of the tested nanomaterials. A CN628 elemental analyzer (LECO Corporation, St. Joseph, MI, USA) was selected to measure total carbon and nitrogen in photocatalysts’ samples. For non-calcined APTES/TiO_2_ nanomaterials, the certified ethylenediaminetetraacetic acid (EDTA) standard (Elemental Microanalysis Ltd., Okehampton, UK) containing 41.04 ± 0.15 wt.% of carbon and 9.56 ± 0.11 wt.% of nitrogen was utilized to prepare the calibration curves. While for calcined APTES-modified TiO_2_, a certified soil standard (Elemental Microanalysis Ltd., Okehampton, UK) containing 0.043 wt.% ± 0.01 of nitrogen and 0.46 wt.% ± 0.15 of carbon, was used. The zeta potential values were measured using a ZetaSizer NanoSeries ZS (Malvern PANalytical Ltd., Malvern, UK) instrument. In order to calculate the Brunauer–Emmett–Teller (BET) specific surface area and pore volume, the low-temperature nitrogen adsorption–desorption measurements at 77 K were performed on the QUADRASORB evoTM Gas Sorption analyzer (Anton Paar GmbH, Graz, Austria). All materials were degassed for 12 h at 100 °C under a high vacuum prior to measurements to eliminate all remaining contaminants on the tested samples’ surface. The total pore volume (V_total_) was derived from the single-point value from the nitrogen adsorption isotherms at relative pressure *p*/*p*_0_ = 0.99, while micropore volume (V_micro_) was calculated using the Dubinin–Radushkevich method, and the mesopore volume (V_meso_) was derived from the difference between V_total_ and V_micro_. The Hitachi SU8020 Ultra-High Resolution Field Emission Scanning Electron Microscope (Hitachi Ltd., Tokyo, Japan) was used to characterize the surface morphology of synthesized photocatalysts. The spectrophotometer UV-Vis V-650 (JASCO International Co., Tokyo, Japan), fitted with a PIV-756 integrating sphere accessory, allowing measurement of DR spectra (JASCO International Co., Tokyo, Japan), was utilized to investigate the light reflectance abilities of the prepared samples. Spectralon^®^ Diffuse Reflectance Material (Labsphere, New Hampshire, NE, USA) was selected as the reference material. The Tauc transformation was used to calculate the band gap energy (E_g_) [85].

### 3.4. Photocatalytic Activity Measurements

The methylene blue decomposition process under artificial solar light irradiation (radiation intensity of 837 W/m^2^ for 300–2800 nm and 0.3 W/m^2^ for the 280–400 nm regions) was carried out to determine the photocatalytic properties of all the prepared samples. All experiments were conducted in a glass beaker using 0.5 L of dye solution with the initial concentration of 5 mg/L and 0.5 g/L of the appropriate semiconductor. Before irradiation, the suspension was magnetically stirred in light-free conditions for 60 min to establish the adsorption–desorption equilibrium at the photocatalyst–methylene blue interface. Then, the mixture was subjected to artificial solar light radiation, and the total exposure time was 360 min. The absorbance value of methylene blue, from which the dye concentration was calculated, was measured every 60 min using a spectrometer UV-Vis V-630 (Jasco International Co., Tokyo, Japan), at the maximum wavelength of 663 nm. Prior to each measurement, 10 mL of the collected suspension was centrifuged to eliminate the suspended TiO_2_ nanoparticles. The methylene blue decomposition degree was calculated according to the following equation:

(1) $$D=\frac{C_{0}-C_{t}}{C_{0}}\times 100\%$$

where *D* is dye decomposition degree (%), *C*_0_ is the initial concentration of the methylene blue solution after the adsorption process (mg/L), and *C_t_* is the concentration of the dye after illumination for t min (mg/L).

## 4. Conclusions

The effect of solvothermal modification of TiO_2_ via APTES, combined with the heat treatment at 800–1000 °C, on the photocatalytic activity of APTES/TiO_2_ nanomaterials under artificial solar light irradiation, was a novelty of the presented study. The presence of APTES on the surface of TiO_2_ was proved via FT-IR/DRS measurements, nitrogen and carbon analyses, and the EDX mapping. It was noted that the presence of Si and C in the APTES-modified TiO_2_ contributed to the effective inhibition of the anatase-to-rutile phase transformation and the growth of the crystallites size of both polymorphous forms of TiO_2_ during calcination at high temperature. Thus, the calcined APTES/TiO_2_ photocatalysts exhibited higher values of S_BET_ and pore volume compared with unmodified reference samples. Changing the surface charge of modified TiO_2_ from positive to negative after calcination increased the methylene blue adsorption degree. However, due to blocking of active sites on the semiconductor surface by APTES molecules, the adsorption process negatively affected the photocatalytic properties. The calcination process increased the artificial solar light-driven photoactivity of all APTES/TiO_2_ materials. For all three tested APTES concentrations, the best dye decomposition degree was received for nanomaterials calcined at 900 °C due to higher S_BET_ values than materials calcined at 1000 °C and larger number of active sites available on the TiO_2_ surface in comparison with samples heated at 800 °C. Considering the highest photocatalytic activity and economic aspects, TiO_2_-4 h-180 °C-500 mM-Ar-900 °C was found to be the most promising photocatalyst.

## Figures and Tables

**Table 1:** Physicochemical properties of starting TiO_2_, reference samples and APTES-modified TiO_2_ nanomaterials.

Sample Name	S_BET_ [m^2^/g]	V_total_ [cm^3^/g]	V_micro_ [cm^3^/g]	V_meso_ [cm^3^/g]	Anatase in Crystallite Phase [%]	Anatase Crystallite Size [nm]	Rutile in Crystallite Phase [%]	Rutile Crystallite Size [nm]
starting TiO_2_	207	0.370	0.070	0.300	95	14	5	21
TiO_2_-Ar-800 °C	6	0.020	0.002	0.018	1	>100	99	>100
TiO_2_-Ar-900 °C	3	0.008	0.002	0.006	-	-	100	>100
TiO_2_-Ar-1000 °C	4	0.009	0.001	0.008	-	-	100	>100
TiO_2_-4 h-180 °C-100 mM	169	0.226	0.065	0.161	96	15	4	51
TiO_2_-4 h-180 °C-100 mM-Ar-800 °C	70	0.201	0.026	0.175	96	23	4	62
TiO_2_-4 h-180 °C-100 mM-Ar-900 °C	35	0.113	0.041	0.072	87	34	13	>100
TiO_2_-4 h-180 °C-100 mM-Ar-1000 °C	8	0.044	0.003	0.041	6	48	94	>100
TiO_2_-4 h-180 °C-500 mM	124	0.162	0.048	0.114	96	15	4	48
TiO_2_-4 h-180 °C-500 mM-Ar-800 °C	95	0.221	0.038	0.183	96	20	4	58
TiO_2_-4 h-180 °C-500 mM-Ar-900 °C	46	0.192	0.017	0.175	94	30	6	55
TiO_2_-4 h-180 °C-500 mM-Ar-1000 °C	16	0.069	0.006	0.063	12	47	88	>100
TiO_2_-4 h-180 °C-1000 mM	121	0.174	0.049	0.125	96	15	4	51
TiO_2_-4 h-180 °C-1000 mM-Ar-800 °C	104	0.215	0.039	0.176	96	19	4	67
TiO_2_-4 h-180 °C-1000 mM-Ar-900 °C	55	0.166	0.021	0.145	94	30	6	54
TiO_2_-4 h-180 °C-1000 mM-Ar-1000 °C	12	0.046	0.005	0.041	16	45	84	>100

**Table 2:** The zeta potential values, carbon and nitrogen contents, and band gap energy (E_g_) of starting TiO_2_, reference samples, and APTES-modified TiO_2_ nanomaterials.

Sample Name	Zeta Potential δ [mV]	Carbon Content [wt.%]	Nitrogen Content [wt.%]	E_g_ [eV]
starting TiO_2_	+12.8	-	0.18	3.29
TiO_2_-Ar-800 °C	−35.9	-	-	3.03
TiO_2_-Ar-900 °C	−36.7	-	-	3.03
TiO_2_-Ar-1000 °C	−41.3	-	-	3.01
TiO_2_-4 h-180 °C-100 mM	+13.6	2.10	0.79	3.27
TiO_2_-4 h-180 °C-100 mM-Ar-800 °C	−38.5	0.17	0.08	3.27
TiO_2_-4 h-180 °C-100 mM-Ar-900 °C	−45.8	0.08	-	3.21
TiO_2_-4 h-180 °C-100 mM-Ar-1000 °C	−49.1	0.03	-	3.02
TiO_2_-4 h-180 °C-500 mM	+22.8	3.82	1.41	3.27
TiO_2_-4 h-180 °C-500 mM-Ar-800 °C	−47.4	0.26	0.08	3.24
TiO_2_-4 h-180 °C-500 mM-Ar-900 °C	−51.0	0.22	-	3.24
TiO_2_-4 h-180 °C-500 mM-Ar-1000 °C	−41.3	0.11	-	2.97
TiO_2_-4 h-180 °C-1000 mM	+12.1	4.10	1.47	3.27
TiO_2_-4 h-180 °C-1000 mM-Ar-800 °C	−51.6	0.27	0.11	3.23
TiO_2_-4 h-180 °C-1000 mM-Ar-900 °C	−60.0	0.22	-	3.22
TiO_2_-4 h-180 °C-1000 mM-Ar-1000 °C	−54.4	0.08	-	2.95

## Data Availability

The data presented in this study are available on request from the corresponding author.
